# Digital Microfluidics for Nucleic Acid Amplification

**DOI:** 10.3390/s17071495

**Published:** 2017-06-25

**Authors:** Beatriz Coelho, Bruno Veigas, Elvira Fortunato, Rodrigo Martins, Hugo Águas, Rui Igreja, Pedro V. Baptista

**Affiliations:** 1 i3N|CENIMAT, Departamento de Ciência dos Materiais, Faculdade de Ciências e Tecnologia, Universidade NOVA de Lisboa, Campus de Caparica, 2829-516 Caparica, Portugal; bj.coelho@campus.fct.unl.pt (B.C.); bmrveigas@gmail.com (B.V.); emf@fct.unl.pt (E.F.); rfpm@fct.unl.pt (R.M.); hma@fct.unl.pt (H.A.); 2 UCIBIO, Departamento de Ciências da Vida, Faculdade de Ciências e Tecnologia, Universidade NOVA de Lisboa, Campus de Caparica, 2829-516 Caparica, Portugal

**Keywords:** Digital Microfluidics, nucleic acid amplification, point-of-care diagnostics

## Abstract

Digital Microfluidics (DMF) has emerged as a disruptive methodology for the control and manipulation of low volume droplets. In DMF, each droplet acts as a single reactor, which allows for extensive multiparallelization of biological and chemical reactions at a much smaller scale. DMF devices open entirely new and promising pathways for multiplex analysis and reaction occurring in a miniaturized format, thus allowing for healthcare decentralization from major laboratories to point-of-care with accurate, robust and inexpensive molecular diagnostics. Here, we shall focus on DMF platforms specifically designed for nucleic acid amplification, which is key for molecular diagnostics of several diseases and conditions, from pathogen identification to cancer mutations detection. Particular attention will be given to the device architecture, materials and nucleic acid amplification applications in validated settings.

## 1. Introduction

Digital Microfluidics (DMF) is a relatively recent technology for liquid manipulation, which allows the control of discrete droplets on a planar surface, through the use of electric, magnetic, optic or acoustic forces [1,2]. The first DMF device dates back to 1986, when Jean Pesant, Michael Hareng, Bruno Mourey and Jean Perbet submitted a patent describing a device “by means of which it is possible to cause fluid globules to circulate within a capillary space by means of pairs of electrodes establishing capture sites” [3]. Later, in the 2000s, two groups (the Fair group at Duke University [4] and the Kim group at University of California, Los Angeles [5]) rediscovered DMF and disseminated the technique, and demonstrated the impact of this technology.

DMF includes all the standard advantages of conventional microfluidics, namely volume reduction, shorter reaction time, increased process sensitivity and decrease in sample cross-contamination [6,7]. Additionally, the devices can be portable and fully automated [6]. Furthermore, DMF offers additional advantages, such as: (1) precise control over unit droplets, (2) easy integration with measurement techniques, (3) multiplex assay capability and (4) there is no need for propulsion devices.

In this review, we will focus on the integration of DMF-based approaches designed for nucleic acid amplification-based genetic testing. Specifically, the development of DMF devices where droplets are transported on electrode arrays via electric forces, namely electrowetting-on-dielectric (EWOD) at relatively low frequencies (non-dielectrophoretic). Within the plethora of designs and settings, we shall highlight those that constitute major advances in DMF construction and evolution. 

### Digital Microfluidics Configurations

DMF devices are mostly derived from two basic configurations: (1) two-plate (or closed) configuration, in which liquid droplets move between two substrates and (2) one-plate (or open) configuration, in which liquid droplets move over one substrate [1]. In the first configuration, the bottom plate includes a substrate (usually glass) where paths of actuation electrodes are deposited, which in turn are covered by a dielectric layer and a hydrophobic layer, preventing sample adhesion to the electrodes. The top plate is generally a single ground electrode, made of transparent, conductive material, allowing for process monitorization (e.g., droplet transportation and reaction visualization by colour change). A filler medium (typically a low viscosity oil) may be added between plates, to reduce evaporation effects and lower the actuation voltage [8]. The second configuration includes all the elements from the previously described bottom-plate, and also allocates the ground electrodes (unless catenae are used as ground electrodes) [2]. Nowadays, most DMF systems are produced in a closed configuration, since it enables all the fluidic operations (dispensing, merging, mixing and splitting), whereas open configurations only allow moving and merging operations [2]. 

Bottom plates are usually glass, monocrystalline silicon, or printed circuit boards (PCB). Substrate choice is closely related to device architecture, ease of fabrication and production cost [9,10]. Contact electrodes are generally metallic, yet alternative conductors such as Indium–Tin–Oxide and doped silicon have been reported. Commonly used dielectrics are Parylene, SU-8 and Si_3_N_4_. Finally, Teflon^®^ (Chemours, Wilmington, DE, USA) and Cytop^®^ (AGCChem, Exton, PA, USA) are standard choices for the hydrophobic layer [2,11].

## 2. Digital Microfluidics for Nucleic Acid Amplification 

Nucleic acid amplification is one of the most relevant tools in molecular biology, allowing the monitorization of gene expression, quantification of food-borne pathogens, diagnostic of hereditary and infectious diseases, as well as numerous forensic analysis processes [12]. The identification and characterization of nucleic acid sequences has become a pivotal element in molecular diagnostics of numerous diseases, such as cancer and hereditary conditions, and detection of pathogens via their unique molecular signature. Thus, nucleic acid amplification technologies (NAATs) have been developed in a multitude of strategies focusing on low sample volume, high specificity and selectivity, and preferably in a portable format. Among these NAATs, the polymerase chain reaction (PCR) is still the “gold standard” for most applications [13]. However, the need to miniaturization and the emergence of microfluidics has prompted amplification strategies that are simpler, i.e., do not require complex temperature cycling for detection of amplification. Such technologies include isothermal loop amplification (LAMP) and rolling circle (RCA) that may be coupled to label free detection schemes [14,15,16]. Particularly, DMF devices have proved to bring several advantages over standard DNA amplification, namely reagent volume reduction, minimization of the analysis time and the possibility of process automation [17,18,19].

### 2.1. Development of DMF–PCR Platforms

Being the first nucleic acid amplification technique to be developed [20], PCR is today the standard technique for nucleic acid amplification, and its working principles have been widespread across all major molecular biology laboratories. Accordingly, it is also the primary procedure for DMF on-chip amplification found in the literature.

**Figure 1:**
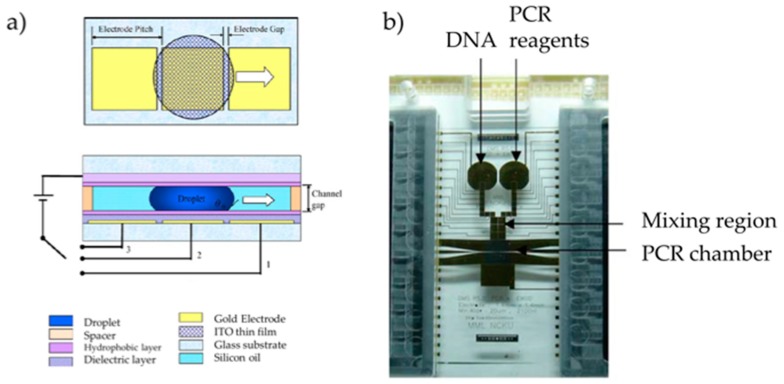
(**a**) Schematic representation of the chip, both in top view and cross-section; (**b**) Photograph of the chip, where the different active regions are visible. Temperature control is based on two platinum heaters, which also provide temperature sensing. Adapted from original in reference [17].

The first group to achieve DNA amplification in DMF platforms was the Chang group [17], by amplifying a detection gene for the Dengue II virus via PCR. For this, a two-plate DMF device was developed, where the top-plate was composed of glass covered with an Indium–Tin–Oxide (ITO) ground electrode, which in turn was covered with a Teflon^®^ hydrophobic layer. The bottom plate included a glass substrate, where Au electrodes were deposited and then covered by a Si_3_N_4_ dielectric layer (0.15 µm) and Teflon^®^. Top and bottom plates were separated by 370 µm, and silicone oil was used as a filler. Two separate regions are included, one for the sample DNA template, and another for the PCR mix reagents (Figure 1).

The described DMF platform was able to successfully amplify a sequence of the Dengue-II virus (511 bps) in 55 min, with a total sample volume of 15 µL. Additionally, this approach allowed for low droplet actuation (12 V_RMS_ at 3 kHz) and PCR chamber operation voltages (9 V_DC_).

**Figure 2:**
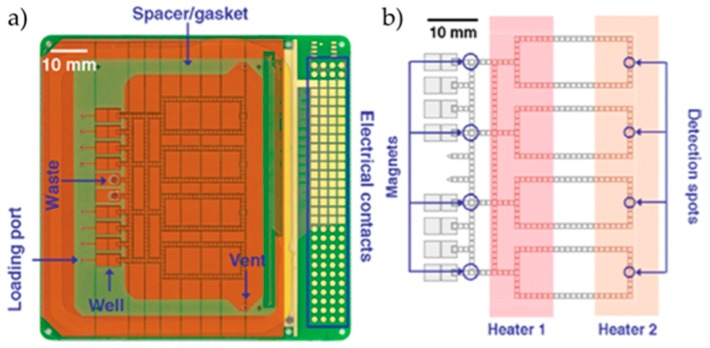
(**a**) Photograph of an assembled digital microfluidics (DMF) chip; (**b**) Schematic of the DMF chip, evidencing the heating system, as well as the optical detection spots. Adapted from original in reference [21].

A partnership between Advanced Liquid Logic Inc. (recently acquired by Illumina) and the Duke University introduced multiplexing in DMF systems, as well as several new improvements in architecture. In this work, Hua et al. developed a fully integrated system, comprised of a control/detection section and a disposable sample processing section (Figure 2) [21].

Based on the closed two-plate format, this improved DMF cartridge chip included 4 detection zones for real-time reaction tracking, allowing for multiplex sample analysis. With this DMF platform, the Hua group performed real-time PCR detection of methicillin-resistant *Staphylococcus aureus* (MRSA), *Mycoplasma pneumoniae* and *Candida albicans*. Amplification was possible using just one copy of DNA template (MRSA genomic DNA). Optimization of PCR step times was pursued, allowing for the reduction of the overall reaction time to 18 min, with no significant changes in performance (threshold cycle and final reaction product). Also, multiple PCR processes were performed simultaneously, and successful detection of MRSA and *M. pneumoniae* was possible.

**Figure 3:**
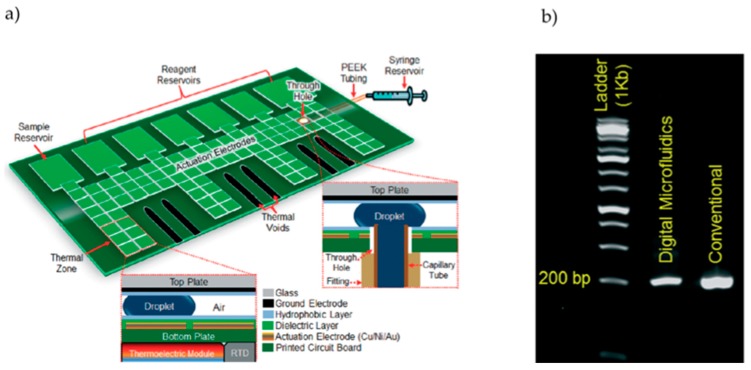
Two-plate air-matrix DMF platform. (**a**) Air-filled DMF platform, featuring a droplet refill system. The primary regions of the device are highlighted, such as actuation electrodes and reagent reservoirs, heating areas (temperature control relies on thermoelectric modules and resistive temperature detectors placed under the printed circuit boards (PCB) substrate) and platform-refill system connections. (**b**) Electrophoretic analysis of on-chip versus off-chip PCR products for M13mp18 bacteriophage DNA, in comparison with a DNA ladder. Adapted from original in reference [22].

As mentioned, DMF systems usually rely on a two-plate architecture, resorting to fillers to help prevent evaporation, one of the key issues in low-volume reactions. However, Jebrail et al. propose a simple approach to use a two-plate air-matrix DMF platform that prevents evaporation on PCR reactions, by refilling reaction droplets with pre-heated solvents whenever necessary [22]. This DMF platform consists of a PCB bottom-plate with Cu/Ni/Au electrodes and reservoirs, covered by a solder mask dielectric layer and a Teflon^®^ hydrophobic layer, and includes a hole connecting the platform to a syringe, which contains refill solvent, through a tube (Figure 3a). Droplet actuation was achieved with 80–100 V_RMS_, at 18 kHz. The DMF chip allowed for PCR-amplification of 200 bp M13mp18 bacteriophage DNA in approximately 45 min, for a final reaction volume of 1.5 µL. During the PCR reaction, 33 refill droplets of solvent with 0.5 µL were added to the reaction droplet, as to revert volume loss due to evaporation. PCR was successfully achieved, yet with lower efficiency than the bench-top counterpart (Figure 3b).

Recently, Norian et al. demonstrated a complementary metal–oxide–semiconductor (CMOS) based DMF device for DNA amplification in point-of-care (POC) diagnostics by real-time PCR [23]. The device was assembled on a ball-grid-array (BGA), in which SU-8 polymer was used to define the droplet manipulation region, and actuation electrodes were coated with Parylene C dielectric, covered by a Teflon^®^ layer, to ensure the hydrophobicity of the bottom plate. This work shows the introduction of flexible components, namely an ITO-coated polyethylene naphthalate (PEN) top plate. The device design includes four reservoirs to store primers, DNA template and PCR reagents, from which 1.2 nL droplets may be retrieved. For actuation, a charge pump voltage converter is able to convert the 3.3 V device power supply into 90 V applied to electrodes to induce an ON state. Real-time optical detection is performed by an integrated Geiger-mode single-photon avalanche diode (SPAD), located at a particular detection pixel in which a window was opened, as to allow the passage of light from an external laser. Several preliminary experiments were performed to evaluate the device, namely temperature calibration, detection performance (nM concentration scale), device lifetime and droplet evaporation (rate of evaporation without filler was found to be 20 pL·s^−1^ for a droplet of 1.2 nL). This chip was applied to the PCR amplification of a 364 bp sequence from *Staphylococcus aureus* and allowed amplification of just one copy of template DNA in a 1.2 nL droplet. 

**Figure 4:**
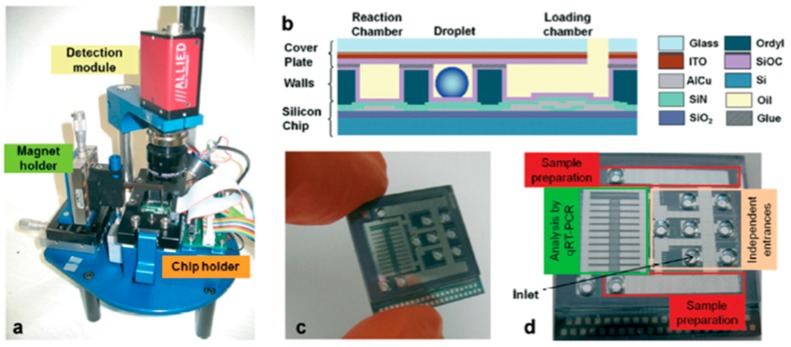
DMF device assembly: (**a**) Additional instrumentation for the DMF chip control; (**b**) Chip cross section, evidencing all the constituting layers; (**c**,**d**) Photographs of the actual DMF chip, showing the most relevant chip regions. Adapted from original in reference [24].

Rival et al. [24] recently presented a DMF platform that allows complete cell expression analysis, from cell lysis to real-time nucleic acid amplification. This two-plate configuration device is produced on a silicon wafer, over which an SiO_2_ insulating layer and Ti/AlCu electrodes were deposited, followed by an Si_3_N_4_ dielectric layer and an SiOC hydrophobic coating. The ITO-covered glass top plate contained sand-blasted holes for reagent and sample insertion, and was separated from the bottom plate by a 100 µm Ordyl^®^ spacer. This DMF platform is accompanied by a bench-top instrument which allows for temperature control (Peltier module), reaction analysis (camera for fluorescence detection after sample excitation with a light emitting diode, (LED)) and droplet actuation (Figure 4). With this platform, cell lysis and subsequent mRNA capture (assisted by magnetic beads) are performed in line, reducing cross contamination probability and greatly improving reproducibility. Platform validation was performed for the detection of the *c-MYC* gene allowing a limit of detection of just one cell, while using a 20-fold volume reduction compared to the traditional benchtop protocols. Furthermore, 10 cells were submitted to the mRNA extraction and amplification procedures, and the real-time fluorescence analysis revealed two distinct Ct values, suggesting sub-populations in the tested sample.

**Figure 5:**
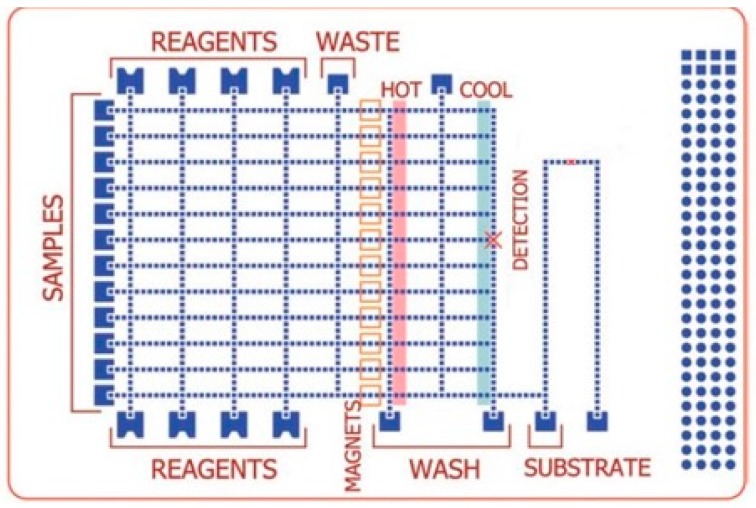
Schematic of the in-house fabricated DMF device for DNA amplification. Top glass plate included drilled ports for sample insertion/withdrawal, and both plates were spaced via a 185 µm polymer film. Adapted from original in reference [9].

Sista et al. [9] developed a DMF platform for POC testing, including PCR-based amplification of pathogenic DNA, with real-time measurement capability. In this closed platform, the bottom plate consisted of an array of independently addressable control electrodes deposited on a printed circuit board (Figure 5). Optical real-time tracking of the PCR reaction was made by using a miniature in-house designed fluorimeter, which consisted of an LED and a photodiode aligned with the detection spot. The design also included a heating system made by two aluminium heating bars placed underneath the platform. A considerable reduction in reaction time, down to 12 min was achieved. Furthermore, it was demonstrated that a single copy of DNA template is enough to achieve amplification in this DMF platform.

Fouillet and co-workers successfully demonstrated the use of a DMF-based chip for single nucleotide polymorphism genotyping in human placental DNA samples [25]. The group developed a closed configuration device, with a polycarbonate top plate, coated with ITO and Teflon^®^. The bottom plate allocated Au electrodes coated with a dielectric layer of Si_3_N_4_ and a hydrophobic Teflon^®^ layer. The cartridge is divided into three regions: (1) loading of reagents; (2) storage section, where intermediate reactions may occur; and (3) channels for droplet actuation. Droplets are actuated at 60 V_RMS_ and 3 kHz. Finally, temperature control was achieved using a Peltier module placed under the chip for fast thermocycling capability. This approach allowed amplification of a minimum of 10 DNA strands, achieving single nucleotide specificity in a total reaction volume of 64 nL. Optical detection was performed by a standard fluorescence microscope using an intercalating dye. This group also developed other interesting DMF chip designs for additional biological processes and applications [26]. 

#### DMF–PCR Platform Validation 

Efforts have been made towards the validation of DMF-based platforms for further development of cost-effective diagnostic methodologies. The first publication showing the validation of a DMF-based platform with clinical samples was reported by Wulff-Burchfield et al., who resorted to DMF-based DNA amplification for the detection of *Mycoplasma pneumoniae* in 59 clinical samples [27]. In this study, the performance of an Advanced Liquid Logic DMF chip was compared to conventional real-time PCR amplification techniques. As a result, successful amplification was achieved for 3.2 µL and the reaction time was reduced by 3-fold, while producing similar results to those attained via bench-top reaction, with 67% sensitivity and 100% specificity.

An Advanced Liquid Logic DMF platform was also employed by Schell et al. [28] for the detection of *Candida albicans* in clinical blood samples. This group performed real-time PCR on 16 blood samples from patients infected with *C. albicans* (1 mL each) and concluded that the PCR-based diagnostic overall performance of on-chip reactions differed from the conventional method, yet the combination of both methods yielded 94% sensitivity. Furthermore, the DMF–PCR approach lowers both reaction time and cost.

### 2.2. DMF—Conventional Microfluidics Hybrid Devices

Another innovative approach is the integration of channel-based conventional microfluidics (CMF) with DMF, creating the so-called hybrid devices. These devices were first developed to fully integrate column separation processes (possible with CMF) with all the steps required for pre-separation (possible with DMF) [29,30]. However, DMF–CMF hybrid devices have also been applied for PCR amplification protocols, either to constrain droplet movement [31] or facilitate reagent injection and heated reactions [32]. 

Ugsornrat et al. developed a hybrid device where fluids are constrained to a PDMS (polydimethylsiloxane) microchannel, yet droplets are still controlled by electrode addressing [31]. Briefly, Cr/Au electrodes were deposited on a glass substrate, and covered with a layer of SiO_2_, which in turn was covered by a hydrophobic layer (Teflon^®^). The glass cover plate included an ITO layer acting as ground electrode, as well as three different serpentine-shaped Cr/Au microheaters, for precise control of denaturation, annealing and extension temperatures in a reaction volume of 25 µL, where optimized usage of mineral oil prevented evaporation beyond standard operating conditions.

**Figure 6:**
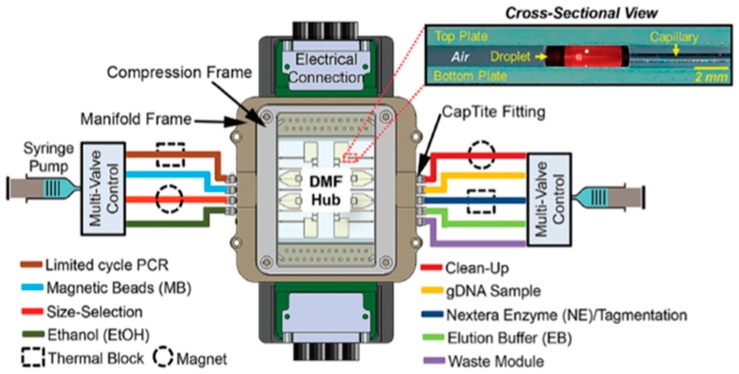
Schematic representation of the DMF platform proposed, evidencing the location of all the reagents required for DNA sequencing. The gap between plates was maintained at either 185 µm or 400 µm, according to the intended droplet volumes, and no filler medium was added. Adapted from original in reference [32].

Kim et al. have developed a DMF platform combining PCR amplification and capillary microfluidics, for DNA sequencing [32]. This device fully integrates nucleic acid amplification (usually required as a pre-sequencing step) with the sequencing procedure itself, unlike other DMF-based sequencing platforms [33,34]. The device is divided into two segments: (1) capillary tubes for reagent insertion on the DMF hub and heated reactions (PCR included) and (2) a DMF hub for reagent mixing and room-temperature reactions, connected to the capillary tubes. The DMF hub consisted of a two-plate configuration with 40 electrodes and 8 larger reservoirs (Figure 6). With this platform, Kim et al. achieved successful PCR amplification of human genomic DNA retrieved from blood cells (previously tagged), in about 13 min, for just 2.8 µL, which further enabled the production of a human DNA library.

### 2.3. DMF for Isothermal Nucleic Acid Amplification

Even though PCR is the most widely known nucleic acid amplification methodology, the three elevated temperatures needed for thermal cycling add complexity to this technique, and contribute to high energy requirements and sophisticated equipment (e.g., thermocyclers). Thus, DNA/RNA amplification research has been progressing towards isothermal amplification schemes, enabling low-cost, low-temperature nucleic acid amplification. Recently, isothermal amplification has also been integrated in DMF devices, envisioning ready-to-use POC molecular diagnostics. 

**Figure 7:**
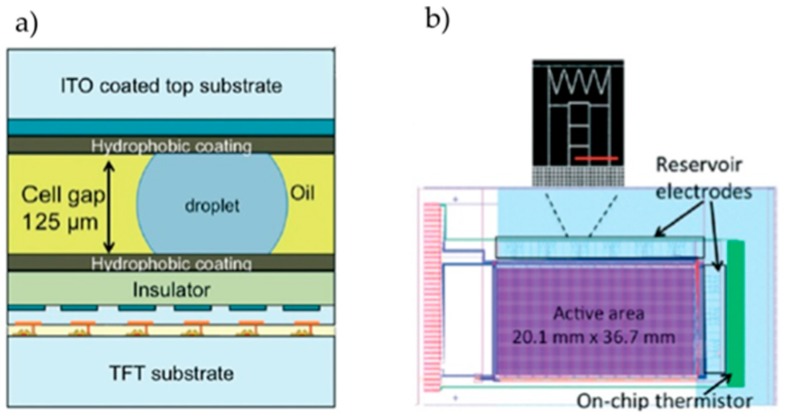
(**a**) Cross-section of the DMF platform, evidencing the several materials that comprise the chip; (**b**) Schematic top view of the chip, evidencing the reservoirs and the temperature sensor. Adapted from original in reference [18].

Kalsi et al. propose an active matrix-based DMF platform for the detection of antibiotic-resistance factors in *Escherichia coli* bacteria (*CTX-M* gene), through isothermal recombinase polymerase amplification (RPA) [18]. This two-plate configuration platform relies on a thin film transistor (TFT) backplane, which controls ITO electrodes for droplet actuation and capacitance sensors able to distinguish between the presence or absence of a droplet, allowing real-time droplet monitoring. The backplane is covered with an Al_2_O_3_ insulator, as well as a disposable Cytop^®^ hydrophobic layer, and the ITO-coated glass top plate is also covered by these two layers (Figure 7). The top plate acts simultaneously as ground electrode and Joule heater (4 W dissipation power), being connected to an off-chip thermistor and a Proportional Integral–Derivative (PID) control system. A Mylar spacer defines a specific distance between electrodes, and droplets are manipulated in an n-Dodecane filler. EWOD actuation voltage is of about 20 V and optical detection is achieved in real-time by fluorescence measurements of a Cy5 label added to the sample. Successful amplification of the gene was possible for a minimum 270 nL, with a limit of detection of about 1 single template copy, in 10 to 15 min. 

Another example of isothermal nucleic acid amplification performed on a DMF chip is provided by Kühnemund et al., to detect very low concentrations of *P. aeruginosa* DNA by rolling circle amplification (RCA) followed by circle-to-circle amplification (C2CA), assisted by magnetic microparticles (MMP) [19]. The amplification protocols are performed on a two-plate configuration chip, where the bottom plate included Cr electrodes covered by Parylene C and Teflon^®^. Two top plate configurations were developed, as to study the influence of the top plate thickness on MMP extraction: ITO-covered glass, covered with a Teflon^®^ layer and Au-covered glass with a top Teflon^®^ layer. Tape was used as spacer, and droplets were actuated by 120 V_AC_, at 1 kHz. Heating was performed by placing the chip on a thermocycler plate. This device allows detection of 1 aM of *P. aeruginosa* DNA in only 60 min, for an RCA reaction temperature of 37 °C and a reaction volume of 5 µL. 

## 3. Alternatives to EWOD for DMF-Assisted Nucleic Acid Amplification

Even though EWOD is the standard method for controlling droplet motion in DMF systems, alternative techniques have been developed, mainly based on magnetic, optic or even acoustic forces. Non-EWOD DMF platforms have been explored for a variety of chemical and biological procedures, namely PCR-based DNA amplification for POC diagnostics. 

Magnetically-assisted DMF platforms for PCR amplification have been widely explored at Johns Hopkins University [35,36,37], and rely on the addition of superparamagnetic particles to the droplets, which in turn are manually controlled by a magnet placed under the substrate and allow droplet motion. Zhang et al. [37] developed a DMF platform for biomarker detection from human blood samples, and pathogen detection from biological samples (*E-coli* bacteria as proof-of-concept). This DMF platform successfully implemented all steps required for target detection, from solid phase DNA extraction to real-time PCR or helicase dependent amplification (HDA), thus proving to be a suitable platform for POC applications. 

DMF devices based on magnetic forces typically do not enable the full spectrum of fluidic operations, namely droplet dispensing and splitting. Zhang et al. [36] surpassed this problem by creating surface energy traps to which droplets bind, thus allowing droplet separation. Droplet splitting enabled multiplex detection of three different biomarkers (*TP53*, *HER2* and *RSF1*) from human blood samples, and once again the entire procedure (from sample to result) was performed on the DMF platform.

Furthermore, Chiou et al. [35] created a fully automated platform for sample-to-result detection of the *KRAS* (Kirsten rat sarcoma viral oncogene homolog) biomarker in human blood, which relies on an electromagnet for transport of the sample droplet through several reaction sports, separated by strictures. 

Apart from human blood, saliva has also been used as a sample by Pipper et al. [38], to detect the avian influenza virus H5N1 on a magnetically-assisted DMF platform, by real-time PCR. This device also performed all the steps required for detection, including RNA solid-phase extraction from the biological sample.

Finally, surface acoustic waves (SAW) and photo-actuation have also emerged as alternatives to EWOD for droplet actuation in DMF platforms for nucleic acid testing. Using SAWs allows low power actuation, however, SAW-mediated devices are still not cost-effective, due to the excessive cost of piezoelectric materials. Photo-actuation eliminates the need for electrodes, therefore simplifying the production process, however, for low-volume droplet actuation (nL scale), expensive laser equipment may be required [39,40,41].

## 4. Future Prospects

DMF development has been gaining momentum strongly focused on nucleic acid amplification, which is at the basis of genetic testing, and plays a fundamental part in POC applications. Joining DMF and nucleic acid amplification represents a major step in reinventing the paradigm of molecular diagnostics, bringing it closer to actual POC.

So far, several DMF platforms have been developed for DNA/RNA amplification, mainly resorting to PCR, the standard technique in genetic testing (Table 1). Real-time reaction monitoring is now a standard requirement, and a variety of target samples were tested, from biological samples to device validation using clinical samples. Others have focused on digital PCR approaches for increased sensitivity in mutation analysis [42,43]. However, efforts have been made to pursue isothermal amplification methodologies, which are intrinsically simpler, with lower energy and equipment requirements than PCR. Recent publications have focused on creating DMF platforms for nucleic acid amplification testing using low cost materials and mass production technologies. Accordingly, simpler reactions lead to simpler devices and economic materials and processes that facilitate commercialization. Indeed, industry is beginning to realize the enormous potential of DMF, and some of the most ground-breaking works in the field have resulted from academia–industry collaborations. More importantly, the first commercial DMF-based devices have been presented, such as Sandia Digital Microfluidics Hub (https://ip.sandia.gov/technology.do/techID=102) or the Illumina NeoPrep sequencing platform (https://www.illumina.com/systems/neoprep-library-system.html).

**Figure 8:**
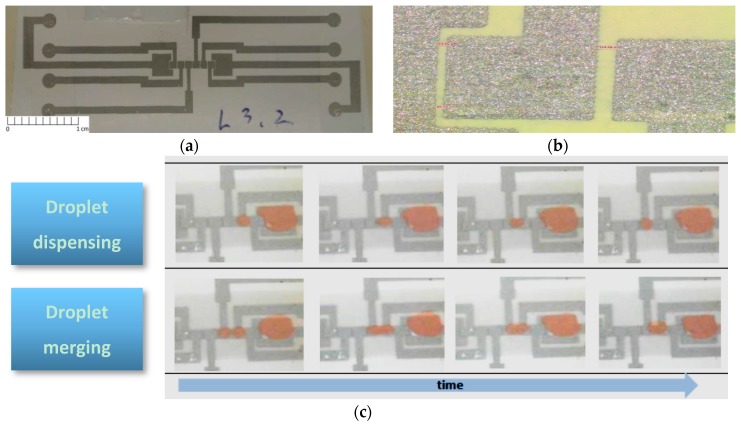
(**a**) DMF device screen-printed on paper substrate; (**b**) Zoom view of the printed electrodes; (**c**) Droplet movement on a paper-based DMF device. Adapted from original in reference [52].

Nevertheless, we strongly believe that DMF for genetic testing will exponentially evolve in the next years, when simulation and control studies (already developed) meet actual platforms, thus empowering fully automated processes, where samples are simply inserted in the platform, and final results are obtained a few minutes later. Furthermore, considering previous experience from our group [44,45,46,47,48,49], we believe that DMF will evolve towards fully disposable devices, with an even lower production cost, resorting to paper-based substrates and printing technologies, such as inkjet [50,51] or screen-printing (Figure 8) [52]. To conclude, in a near future, no specialized laboratory technicians will be required to prepare the samples, perform the assays or read the results, and testing platforms will be portable, low-cost and disposable, thus enabling the true ideal of POC: devices available anytime, anywhere, for everyone.

## Figures and Tables

**Table 1:** Summary of DMF platforms for nucleic acid amplification. ITO—Indium–Tin–Oxide; PCB—Printed Circuit Board; PEN—Polyethylene Naphthalate; qRT-PCR—Quantitative Real-Time Polymerase Chain Reaction; SNP—Single Nucleotide Polymorphism; POC—Point-of-care; RCA—Rolling Circle Amplification; C2CA—Circle-to-circle Amplification; RPA—Recombinase Polymerase Amplification; MRSA—methicillin-resistant *Staphylococcus aureus* (MRSA); *M pneumoniae*—*Mycoplasma pneumoniae*; *C. albicans*—*Candida albicans; E. coli*—*Escherichia coli; P. aeruginosa*—*Pseudomonas aeruginosa.*

	Application	Reaction Volume	Actuation Voltage	Dielectric Material	Hydrophobic Material	Electrode Material	Substrate Material	Filler	Reference
DMF–PCR platforms	Dengue II virus detection	1.46 µL	12 V_RMS_ (3 kHz)	Si_3_N_4_	Teflon^®^ AF	Au	Glass	Silicone oil	[17]
	SNP genotyping	64 nL	60 V_RMS_ (3 kHz)	Si_3_N_4_	Teflon^®^ AF	Au	Glass	Silicone oil	[25]
	POC testing, MRSA, *S. aureus* and *C. albicans* detection	600 nL	-	Parylene C	Teflon^®^ AF	Cr	PCB	Hexadecane	[9]
	MRSA, *S. aureus*, *M. pneumoniae* and *C. albicans* detection	Variable	-	-	-	-	PCB	Hexadecane/silicone oil	[21]
	*S. aureus* detection	1.2 nL	70 V–250 V	Parylene C	Teflon^®^ AF	-	-	n-dodecane	[23]
	Cell genetic expression analysis	Variable	48 V (3 kHz)	Si_3_N_4_	SiOC	Ti/AlCu	Silicon wafer	Silicone oil	[24]
	Bacteriophage M13mp18 detection	1.5 µL	80–100 V_RMS_ (18 kHz)	Solder mask	Teflon^®^ AF	Cu/Ni/Au	PCB	Air	[22]
Validation of DMF–PCR platforms	*C. albicans* detection on human blood	Variable	-	-	-	-	PCB	Hexadecane/silicone oil	[27]
	*M. pneumoniae* detection on human nasopharyngeal wash	Variable	-	-	-	-	PCB	Hexadecane/silicone oil	[28]
Hybrid platforms	DNA amplification (only the primers are described)	25 µL	-	SiO_2_	Teflon^®^ AF	Cr/Au	Glass	Silicone oil	[31]
	Pre-DNA sequencing	2.8 µL	80–90 V_RMS_ (15 kHz)	Parylene C	Teflon^®^ AF	ITO	Glass	Air	[32]
DMF platforms for isothermal amplification	*CTX-M* gene detection in *E. coli* bacteria	Minimum 270 nL	20 V	Al_2_O_3_	Cytop^®^	ITO	Glass	n-dodecane	[18]
	*P. aeruginosa* detection	5 µL	120 V_AC_ (1 kHz)	Parylene C	Teflon^®^ AF	Cr	Glass	Vapor-Lock oil	[19]
